# Functional Analysis of the NLR Gene *YPR1* from Common Wild Rice (*Oryza rufipogon*) for Bacterial Blight Resistance

**DOI:** 10.3390/genes16111321

**Published:** 2025-11-02

**Authors:** Wang Kan, Zaiquan Cheng, Yun Zhang, Bo Wang, Li Liu, Jiaxin Xing, Fuyou Yin, Qiaofang Zhong, Jinlu Li, Dunyu Zhang, Suqin Xiao, Cong Jiang, Tengqiong Yu, Yunyue Wang, Ling Chen

**Affiliations:** 1 College of Plant Protection, Yunnan Agricultural University, Kunming 650224, China; mr_kanwang@163.com; 2 Biotechnology and Germplasm Resources Institute, Yunnan Academy of Agricultural Sciences/Yunnan Provincial Key Laboratory of Agricultural Biotechnology/Key Laboratory of Southwestern Crop Gene Resources and Germplasm Innovation, Ministry of Agriculture, Kunming 650205, China; czquan-99@163.com (Z.C.);; 3 National Wild Rice Germplasm Resources Nursery, Kunming 650224, China

**Keywords:** common wild rice, white leaf blight, expression pattern, induced expression, tissue-specific expression, CRISPR/Cas9, NLR gene, disease resistance breeding

## Abstract

**Background/Objectives:** Bacterial blight (BB) represents one of the most devastating diseases threatening global rice production. Exploring and characterizing disease resistance (R) genes provides an effective strategy for controlling BB and enhancing rice resilience. Common wild rice (*Oryza rufipogon*) serves as a valuable reservoir of genetic diversity and disease resistance resources. In this study, we identified and functionally characterized a novel NLR gene, *YPR1*, from common wild rice (*Oryza rufipogon*), which exhibited significant spatial, temporal, and tissue-specific expression patterns. **Methods:** Using a combination of conventional PCR, RT-PCR, bioinformatics, transgenic analysis, and CRISPR/Cas9 gene-editing approaches, the full-length *YPR1* sequence was successfully cloned. **Results:** The gene spans 4689 bp with a coding sequence (CDS) of 2979 bp, encoding a 992-amino acid protein. Protein domain prediction revealed that YPR1 is a typical CNL-type NLR protein, comprising RX-CC_like, NB-ARC, and LRR domains. The predicted molecular weight of the protein is 112.43 kDa, and the theoretical isoelectric point (pI) is 8.36. The absence of both signal peptide and transmembrane domains suggests that YPR1 functions intracellularly. Furthermore, the presence of multiple phosphorylation sites across diverse residues implies a potential role for post-translational regulation in its signal transduction function. Sequence alignment showed that *YPR1* shared 94.02% similarity with *Os09g34160* and up to 96.47% identity with its closest homolog in the NCBI database, confirming that *YPR1* is a previously unreported gene. To verify its role in disease resistance, an overexpression vector (Ubi–YPR1) was constructed and introduced into the BB-susceptible rice cultivar JG30 via *Agrobacterium tumefaciens*-mediated transformation. T_1_ transgenic lines were subsequently inoculated with 15 highly virulent *Xanthomonas oryzae* pv. *oryzae* (*Xoo*) strains. The transgenic plants exhibited strong resistance to eight strains (YM1, YM187, C1, C5, C6, T7147, PB, and HZhj19), demonstrating a broad-spectrum resistance pattern. Conversely, CRISPR/Cas9-mediated knockout of *YPR1* in common wild rice resulted in increased susceptibility to most *Xoo* strains. Although the resistance of knockout lines to strains C7 and YM187 was comparable to that of the wild type (YP^WT^), the majority of knockout plants exhibited more severe symptoms and significantly lower *YPR1* expression levels compared with YP^WT^. **Conclusions:** Collectively, these findings demonstrate that *YPR1* plays a crucial role in bacterial blight resistance in common wild rice. As a novel CNL-type NLR gene conferring specific resistance to multiple *Xoo* strains, *YPR1* provides a promising genetic resource for the molecular breeding of BB-resistant rice varieties.

## 1. Introduction

Rice bacterial blight (BB), caused by *Xanthomonas oryzae* pv. *oryzae* (*Xoo*), is one of the most destructive diseases threatening rice production in China and other major rice-growing regions worldwide. It leads to severe yield losses and poses a major challenge to global food security. Among various control strategies, the exploration and utilization of effective resistance (R) genes remain the most sustainable and economical approach for managing this disease. To date, approximately 48 BB resistance genes have been identified and mapped across the 12 chromosomes of rice. Most of these genes are located on chromosomes 4 and 11, whereas no resistance genes have been reported on chromosomes 9 and 10 [1]. Notably, nearly 85% of these R genes have been derived from cultivated rice, with relatively few discovered in wild rice species [2]. The genus *Oryza L.* consists of 24 species, including two cultivated species and 22 wild species. Wild rice exhibits extensive genetic diversity in traits such as disease resistance, pest resistance, cold tolerance, and morphological variation (tall, dwarf, creeping, or erect growth). Among these, stress-related traits such as disease and pest resistance are particularly valuable for crop improvement [3]. Because of its close evolutionary relationship with cultivated rice (*Oryza rufipogon* Griff.) [4], common wild rice is an important reservoir of elite alleles that can be used in modern breeding. Common wild rice (*Oryza rufipogon*), considered one of the origins of Asian cultivated rice [5], harbors numerous valuable genes, including *SHA1* (seed shattering) [6], *OsLG1* (loose panicle) [7], *LABA1* (long awn) [8], *PROG1* (prostrate growth) [9], and *Xa47* (bacterial blight resistance) [10,11]. However, the resistance spectrum of Xa47 does not fully account for the broad-spectrum and durable resistance observed in the Yuanjiang common wild rice accession, suggesting the presence of additional, unidentified R genes in this germplasm. Among these, *Xa47* encodes an NLR-type resistance protein that confers bacterial blight resistance; however, its resistance spectrum does not fully account for the broad-spectrum resistance observed in Yuanjiang common wild rice [12]. This observation suggests that additional, yet unidentified, bacterial blight resistance genes may exist in this germplasm, emphasizing the importance of further exploration.

Nucleotide-binding site–leucine-rich repeat (NLR) genes represent one of the largest and most conserved classes of plant disease resistance genes. They play central roles in recognizing pathogen effectors and activating downstream immune signaling [13]. Based on their N-terminal domains, plant NLRs are categorized as Toll/interleukin-1 receptor NLRs (TNLs) or coiled-coil NLRs (CNLs) [14]. The TIR domain mediates specificity and signaling through amino acid polymorphisms [15], while the CC domain facilitates protein–protein interactions [16]. The central nucleotide-binding (NB-ARC) domain acts as a molecular switch in defense activation [17,18], and the leucine-rich repeat (LRR) domain determines recognition specificity by mediating protein–protein interactions and effector perception [19]. NLR genes are evolutionarily dynamic, frequently undergoing duplication, recombination, and mutation to generate novel alleles and broaden pathogen recognition spectra [20]. Nevertheless, their structural and functional diversity across species remains complex, and many NLR-mediated resistance mechanisms are still poorly understood [21]. Consequently, isolating and characterizing novel NLR genes provides essential theoretical and genetic foundations for improving rice disease resistance through molecular breeding.

Comparative genomic analyses have revealed extensive NLR gene repertoires in rice. The number of NLR-coding genes ranges from 419 to 511 in cultivated rice and from 159 to 669 in wild rice [22]. However, the functional characterization of these genes is still at an early stage. In our previous study, the expression profiles of NLR genes on chromosome 9 of *Oryza sativa* cv. Nipponbare was analyzed in common wild rice. Among them, *Os09g34160* exhibited a strong and specific induction by *Xoo*, with higher expression levels in leaves than other NLR genes on the same chromosome. This finding suggested that *Os09g34160* may play an important role in the resistance of common wild rice to bacterial blight [23].

To further investigate its function, the *Os09g34160* homolog from common wild rice was isolated and designated *YPR1*. Using molecular cloning, bioinformatics analysis, transgenic overexpression, and CRISPR/Cas9-mediated gene editing, we analyzed the structural characteristics and resistance function of *YPR1*. The results demonstrated that *YPR1* encodes a novel CNL-type NLR protein conferring specific resistance to multiple *Xoo* strains. This study provides new insights into the molecular mechanisms underlying bacterial blight resistance in wild rice and offers valuable genetic resources for the development of BB-resistant rice cultivars.

## 2. Materials and Methods

### 2.1. Materials

Plant material: common wild rice (from Yuanjiang, Yunnan Province), a perennial herb, belongs to the genus Poaceae. It is collected from the Yuanjiang area, Yunnan Province, China, at an altitude of 780 m. Jingang 30 (JG30), an indica cultivated rice, is generally susceptible to bacterial blight. It is used as a receptor material for genetic transformation and an inoculation control material.

Bacterial blight strain: The tested rice bacterial blight strains included 15 strains isolated and purified from the leaves susceptible to bacterial blight in different rice cultivation areas in China, as well as some standard strong pathogenic strains at home and abroad (Table 1).

### 2.2. Methods

#### 2.2.1. Cloning of DNA Sequence of *YPR1* Gene

The young leaves of Yuanjiang common wild rice were frozen in liquid nitrogen and ground into powder. The total DNA of leaves was extracted using the AxyPrepTM Multisource Genomic DNA Miniprep Kit (Axygen, Corning, NY, USA, Catalog No. AP-MN-MS-GDNA-50). Download the upstream and downstream sequences of the *Os09g34160* gene, and use the Primer-BLAST program (https://www.ncbi.nlm.nih.gov/tools/primer-blast/, accessed on 29 October 2025) in the NCBI database to design and amplify the specific primer YPQC-10-F/R for the full-length DNA of the *YPR1* gene (Table 2). PCR amplification was performed in a 50 μL reaction system containing 26 μL of 2× Vazyme Max Master Mix (Vazyme Biotech, Nanjing, China, Catalog No. P711), 10 μmol/L forward primer 2 μL, 10 μmol/L reverse primer 2 μL, 20 ng/μL template DNA 8 μL, ddH_2_O 12 μL; PCR reaction conditions: pre-denaturation at 94 °C for 3 min; 40 cycles of denaturation at 94 °C for 15 s, renaturation at 55 °C for 15 s, extension at 68 °C for 5 min; extending at 68 °C for 10 min. The 5 μL PCR amplification product was subjected to 5 V/cm constant voltage electrophoresis on 2% agarose gel at 5 V/cm. According to the expected fragment size of 5413 bp when designing primers, the gel imaging system was used to observe the correctness and specificity of the amplified fragment. The amplified PCR product was directly sent to Kunming Branch of Beijing Qingke Biotechnology Co., Ltd. (Kunming, China). for sequencing, in order to obtain the DNA sequence of the *YPR1* gene.

#### 2.2.2. Cloning of cDNA Sequence of *YPR1* Gene

The total RNA of common wild rice was extracted by the Eastep Super RNA LS1040 kit (Promega, Madison, WI, USA). According to the DNA sequence of the *YPR1* gene and the coding region (CDS) of the *Os09g34160* gene, the CDS of the *YPR1* gene was predicted. Based on the predicted sequence, the specific primers YP-CDS-F/R (Table 2) were designed and amplified by RT-PCR. The reaction system was as follows: Max Master Mix 25 μL, 10 μmol/L upstream and downstream primers 2.4 μL each; 20 ng/μL template cDNA 6 μL, ddH_2_O 14.2 μL; PCR reaction conditions: pre-denaturation at 94 °C for 3 min; denaturation at 94 °C for 15 s, renaturation at 64 °C for 15 s, extension at 72 °C for 3 min, a total of 40 cycles; 72 °C extension for 10 min. The PCR products were detected and separated by 1% agarose gel electrophoresis. The PCR products of the *YPR1* gene were recovered by a DNA Gel Extraction Kit (Beijing Qingke Biotechnology Co., Ltd. Kunming, China), and the concentration of PCR products was determined by Nandrop2000. According to the steps described in the 5minTM TA/Blunt-Zero Cloning Kit, the PCR product was ligated with the pCE2 TA/Blunt-Zero vector, and the ligation product was transformed into *E. coli* competent DH5α. After the monoclonal was picked up for liquid culture, the recombinant was identified by bacterial liquid PCR, and the positive bacterial liquid containing the target fragment was sent to Beijing Qingke Biotechnology Co., Ltd. (Kunming, China) for sequencing. The sequencing sequence was intercepted using YP-CDS-F/R primers.

#### 2.2.3. Bioinformatics Analysis of YPR1 Gene

The cDNA sequence of the *YPR1* gene was input into BioXM 2.7.1 software for translation, to obtain the amino acid sequence of *YPR1*. The amino acid sequence of YPR1 and Os09g34160 was analyzed by multiple alignment using DNAMAN version 9.0 (Lynnon Biosoft, CA, USA). At the same time, the amino acid sequence of the *YPR1* gene was input into the blastp program of the NCBI database to analyze the homology and similarity of the YPR1 protein with other genes. The molecular weight, isoelectric point, average coefficient of hydrophilicity, and the number of positively and negatively charged amino acids of the deduced amino acid sequence were analyzed using the ProtParam v2.0 online tool in the ExPASy database. NetPhos 3.1 Server was used to predict the potential phosphorylation sites of YPR1 protein. The GOR4 tool was used to predict the protein secondary structure. The protein BLAST tool of the NCBI database was used to analyze the consistency of YPR1 protein and its homologous protein. Multiple sequence analysis was performed by DNAMAN, phylogenetic analysis was performed by MEGA 6.0, and a phylogenetic tree was constructed by Neighbor-joining (NJ) algorithm.

#### 2.2.4. Construction of Ubi-YPR1 Overexpression Lines

The total RNA of Yuanjiang common wild rice was extracted and reverse transcribed into cDNA. RT-PCR amplification was performed using YP-CDS-F/R. The amplified fragment was recovered and ligated with the 5minTM TA/Blunt-Zero Cloning Kit vector provided by Vazyme Company (Nanjing, Jiangsu, China). The ligation product was sequenced to determine the successful ligation, and then the TA-CDS plasmid was obtained. The pCamBIA1305 plasmid was selected as the backbone of the plant expression vector, and it was double digested with XbaI and BstEII. The ubiquitin promoter in the plasmid pJET-Ubi was amplified using the primer Ubi-F/R (Table 2). The lowercase part of Ubi-F is the partial sequence of the digested site on the pCamBIA1305 plasmid, the uppercase part is the 5′ end sequence on the ubiquitin promoter, the lowercase part of Ubi-R is the 5′ end sequence on the *YPR1* CDS, and the uppercase part is the 3′ end sequence on the ubiquitin promoter. The YP-TY-F/R (Table 2) primers were used to amplify the *YPR1* sequence in the plasmid TA-CDS. Among them, the lowercase part of YP-TY-F was the 3′ end sequence on the ubiquitin promoter, the uppercase part was the 5′ end sequence on the *YPR1* CDS, the lowercase part of YP-TY-R was the partial sequence of the digested site on the pCamBIA1305 plasmid, and the uppercase part was the 3′ end sequence on the *YPR1* CDS. After cutting the gel to recover the target fragment, the homologous recombinase provided by Takara was used to construct the Ubi-YPR1 overexpression vector by homologous recombination. The Ubi-YPR1 overexpression vector was transferred into the cultivated rice JG30, which was susceptible to bacterial blight by the Agrobacterium-mediated method [24].

#### 2.2.5. Construction of Cas9-YPR1 Editing Strain

CRISPR-Cas9 was used to edit the *YPR1* gene in Yuanjiang common wild rice [24]. The DNA sequence of the *YPR1* gene was input into the CRISPR-P2.0 online program to search the target sequence containing the PAM (protospacer adjacent motif) site. The target site with a score greater than 0.5 and located at the 5 ′ end of the DNA sequence of the *YPR1* gene was selected as the candidate sgRNA sequence. Based on the sgRNA sequence, the forward and reverse oligo sequences sgRNA-F and sgRNA-R were designed, respectively, and the GGCA sequence was added before the sgRNA-F. The AAAC sequence (Table 2) was added before the sgRNA-R, and the synthesis was commissioned by the Kunming Branch of Beijing Qingke Biological Company (Kunming, China). All primers in this study were synthesized by the company. The synthesized oligodimer was ligated with the entry vector pOs-sgRNA digested by Bsa I, and then transformed into E.coli competent cells Trelief TM 5α by heat shock. The bacterial liquid was obtained by shaking the culture at 37 °C and 200 r/min for 2–3 h. A total of 200 μL of bacterial solution was coated on an LB solid plate containing 50 mg/L kanamycin, and monoclonals were obtained after 12 h of dark culture at 37 °C. The monoclonals were picked up into 5 mL of LB liquid medium containing 50 mg/L kanamycin. The primer Seq-U3 (Table 2) on the pOs-sgRNA vector was used for sequencing verification, and the Kunming Branch of Beijing Qingke Biological Company was entrusted to complete it, and then the pOs-YPR1-sgRNA recombinant vector was obtained. The pOs-YPR1-sgRNA recombinant vector was recombined with the target vector pH-Ubi-Cas9-7, and the reaction product was transformed into E. coli competent cell Trelief TM 5α by the heat shock method. The bacterial liquid was obtained by shaking the culture at 37 °C and 200 r/min for 2–3 h. A total of 200 μL of bacterial solution was coated on LB solid plates containing 25 mg/L streptomycin and spectinomycin, and monoclonals were obtained by dark culture at 37 °C for 12 h. Monoclonals were picked up and cultured in 5 mL LB liquid medium containing 25 mg/L streptomycin and spectinomycin at 37 °C and 200 r/min for 12 h. The culture medium was obtained, and the plasmid was extracted by plasmid extraction kit. Using Seq-U3 and Seq-Cas primers (Table 2), the sequencing was commissioned by the Kunming Branch of Beijing Optico Biological Company (Kunming, China). Finally, the Cas9-YPR1 knockout vector was obtained. The Cas9-YPR1 knockout vector was transferred into Yuanjiang common wild rice materials by the Agrobacterium-mediated method [25].

#### 2.2.6. Identification of Overexpression and Gene Editing Positive Lines

A DNA extraction kit was used to extract DNA from the leaves of T_0_ generation plants, and the Hyg-F/Hyg-R (Table 2) specific primers of the hygromycin resistance gene Hyg-F/Hyg-R were used for PCR amplification to identify positive transgenic seedlings. The reaction system was as follows: Max Master Mix 7.5 μL, 10 μmol/L upstream and downstream primers 1 μL, 20 ng/μL template DNA 2 μL, ddH_2_O 3.5 μ. PCR reaction conditions: pre-denaturation at 94 °C for 3 min; denaturation at 94 °C for 15 s, renaturation at 60 °C for 15 s, extension at 72 °C for 45 s, a total of 35 cycles; 72 °C extension for 10 min. The PCR products were detected by 2.5% agarose gel electrophoresis.

#### 2.2.7. Mutation Site Analysis of Gene Editing Positive Lines

According to the nucleic acid sequences on both sides of the *YPR1* gene editing site, primers YP-YPR1-22-F/R (Table 2) were designed to amplify the target fragment of the mutant plant leaves, and the reaction system was the same as 1.3. PCR reaction conditions: pre-denaturation at 94 °C for 3 min; denaturation at 94 °C for 15 s, renaturation at 55 °C for 15 s, extension at 72 °C for 2 min, a total of 40 cycles; 72 °C extension for 10 min. The 6 μL PCR product was subjected to 2.5% agarose gel electrophoresis at a constant pressure of 5 V/cm, and the gel imaging system was used to image it. The remaining PCR products were sequenced using the corresponding amplification primers, and entrusted to Kunming Branch of Beijing Qingke Biological Company. According to the sequencing results, the mutation type was analyzed. Mutations in the single-peak normal sequencing map are considered to be homozygous mutations, and mutations containing the nested-peak sequencing map are considered to be heterozygous mutations or double-allelic mutations, which are decoded according to the degenerate sequence characteristics of DNA [26].

#### 2.2.8. Determination of YPR1 Expression in Genetic Transformation Lines

The obtained overexpression and gene editing positive lines were selected to detect the expression of the *YPR1* gene, with wild-type JG30 and Yuanjiang common wild rice as controls. The young leaves of the above materials were taken, respectively, and the total RNA was extracted by RNA extraction kit, and the total RNA was reverse transcribed into cDNA by HiScript^®^ III RT SuperMix for qPCR kit (Vazyme Biotech, Nanjing, China). The specific primer Yg-YPR1-F/R (Table 2) of the *YPR1* gene was designed for qPCR detection with *actin* as the internal reference gene. The 20 μL qPCR reaction system was prepared using the ChamQ™ Universal SYBR^®^ qPCR Master Mix kit (Vazyme Biotech, Nanjing, China), that is, 2× qPCR Mix 10 μL, 10 μmol/L upstream and downstream primers 0.4 μL, 10 ng/μL cDNA template 1 μL, ddH_2_O 8.2 μL. Each sample was subjected to three parallel reactions and amplified by a two-step method. The procedure was as follows: pre-denaturation at 95 °C for 60 s; denaturation at 95 °C for 10 s; annealing at 60 °C for 10 s; a total of 40 cycles. The relative expression of the *YPR1* gene was analyzed by the 2^−ΔΔCt^ method.

#### 2.2.9. Resistance Identification of Genetic Transformation Lines to Bacterial Blight

Fifteen strains (C1, C2, C3, C5, C6, C7, C9, Y8, T7147, PXO99^A^, PB, Hzhj19, YM1, YM187, and YJdp-2) were inoculated onto NA plates and cultured in inverted dark at (28 ± 2) °C for 48–72 h. The bacteria on the plate were eluted with sterile distilled water and prepared into a suspension with a concentration of 3 × 108 CFU/mL (OD600nm = 0.5). The genetic transformation lines T0 and T1 mutant plants were inoculated in vivo at the booting stage to analyze their resistance to bacterial blight. The prepared bacterial suspension of bacterial blight was dipped, respectively, and 10 plants of the same variety were inoculated with each strain. Five leaves were selected for each plant, and the leaf tip of 1–3 cm was cut off from each leaf, and the bacterial solution was dipped once for each cut, so it was repeated until the inoculation was completed. After inoculation, they were cultured under natural conditions and the incidence was observed and recorded. After about 15 days of inoculation, when the disease development of the susceptible control material Jingang 30 tended to be stable, three non-damaged leaves with the longest lesion (no pests or other diseases except bacterial blight and mechanical damage) were selected from each plant to measure the length of the lesion. The lesion length of 6 cm was used as the boundary between resistance and susceptibility; that is, the lesion length less than or equal to 6 cm was defined as resistance, and the lesion length greater than 6 cm was defined as susceptibility.

## 3. Results

### 3.1. Cloning of YPR1 Gene

**Figure 1:**
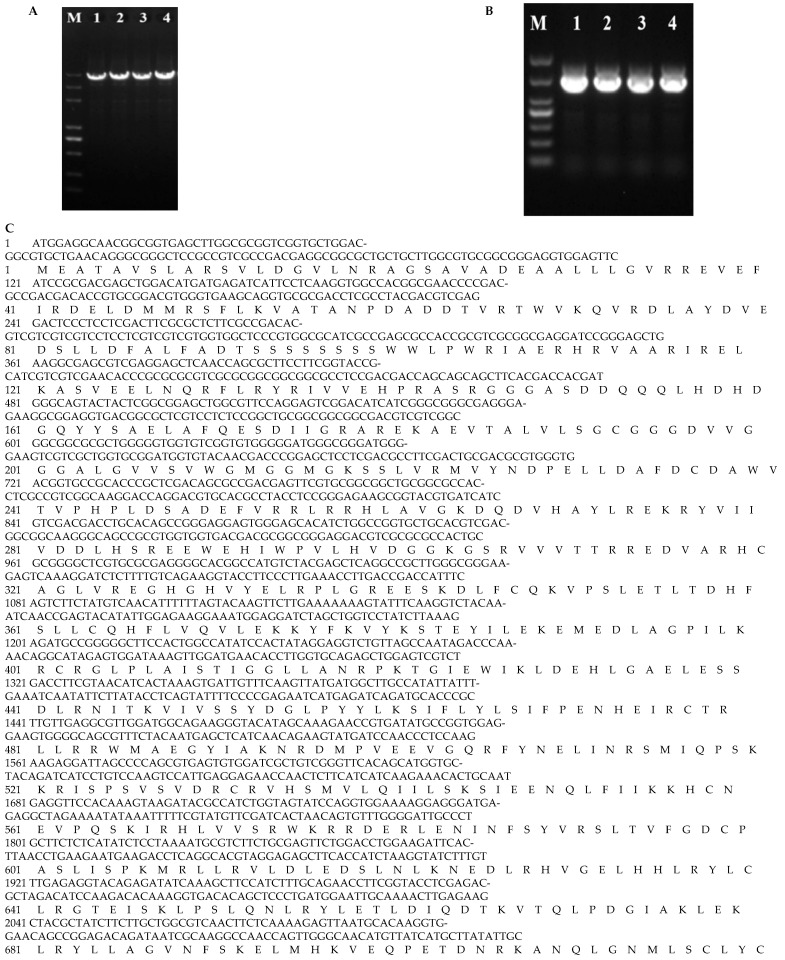

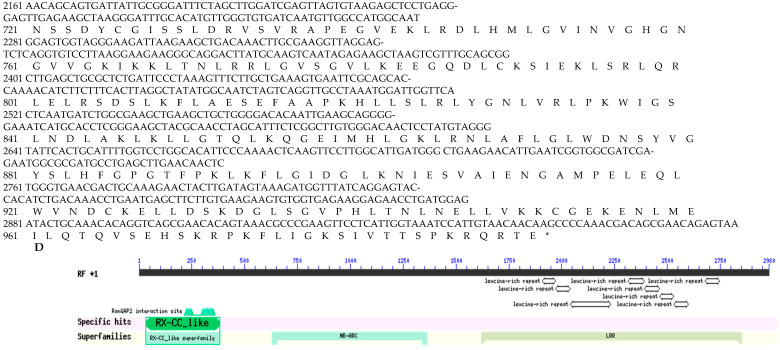
Analysis of the basic characteristics of the *YPR1* gene DNA cloning of *YPR1* gene (**A**). cDNA cloning of *YPR1* gene (**B**). The coding region and amino acid sequence of *YPR1* gene (**C**). Conserved domain of YPR1 protein (**D**). M: DL5000 marker, 5000 bp, 3000 bp, 2000 bp, 1000 bp, 750 bp, 500 bp, 250 bp from top to bottom. 1, 2, 3, and 4 mean Yuanjiang common wild rice sample number.

The DNA sequence and coding sequence (CDS) of the target gene *YPR1* were obtained by homologous cloning using the DNA and cDNA of Yuanjiang common wild rice as templates. The results showed that the DNA sequence of the gene was 4689 bp (Figure 1A), the CDS was 2979 bp (Figure 1B), and it encoded 992 amino acids (Figure 1C). Domain prediction revealed that YPR1 is a typical CNL-type protein, characterized by an N-terminal RX-CC-like domain, a central NB-ARC domain, and a C-terminal LRR domain, an architecture classically associated with intracellular pathogen recognition and immune activation. The conserved domains of the protein were analyzed by online tools. The results showed that the protein had 1 RX-CC-like, 1 NB-ARC superfamily and 11 LRR domains, which was consistent with the conserved domain of the NLR gene family protein. Specific, is a typical CNL-type NLR family protein (Figure 1D).

### 3.2. Analysis of Physicochemical Properties of YPR1 Protein

**Figure 2:**
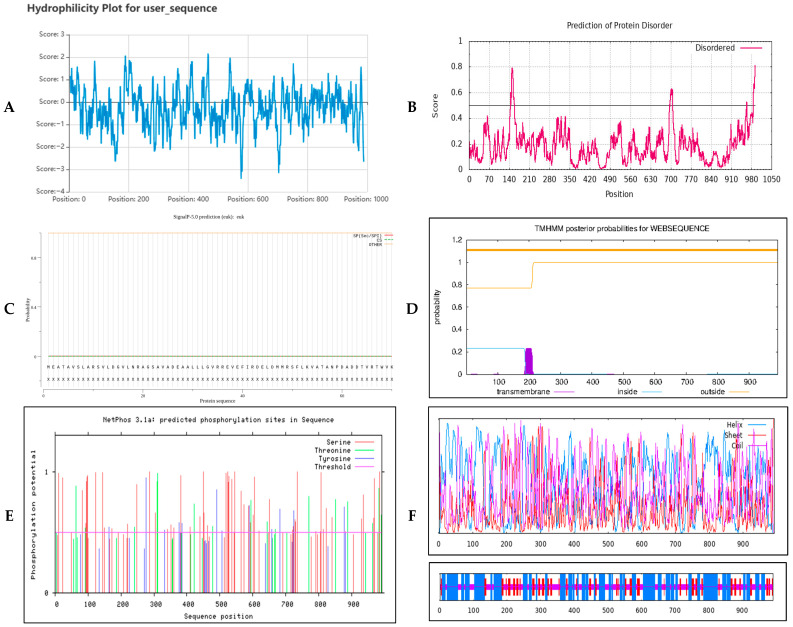
Analysis of physical and chemical properties of YPR1 protein Hydrophobicity prediction of YPR1 protein (**A**). Prediction of the intrinsic disordered region of YPR1 protein (**B**). YPR1 protein signal peptide prediction (**C**). Transmembrane domain prediction of YPR1 protein (**D**). YPR1 protein phosphorylation kinase site prediction (**E**). Protein structure model by residue quality assessment diagram (**F**).

The relative molecular mass of YPR1 protein was 112.43 kDa, the theoretical isoelectric point was 8.36, the fat solubility index was 100.68, the leucine (Leu) content was the highest (13.9%), and the tryptophan (Typ) content was the lowest (1.4%). The total average hydrophilicity coefficient was −0.29, and the total average hydrophobicity coefficient was −0.49 (Figure 2A). There were inherent disordered regions (Figure 2B) in the regions of amino acids around 150,700 and 980, indicating that the protein was a negatively charged, weakly alkaline, and unstable hydrophilic protein. The probability of YPR1 protein having a signal peptide is 0.104% (Figure 2C), and there is no transmembrane region (Figure 2D), indicating that such proteins play a role in synthesis after synthesis, and there is no transport, indicating that it is impossible to be a secreted protein or a protein synthesized in the cytoplasmic matrix that is transported to the organelles to play a role. YPR1 was predicted to contain 91 potential phosphorylation sites, suggesting that its activity may be finely regulated by kinase-mediated signaling pathways, a common regulatory mechanism for NLR proteins, including 61 serine (S) sites, 18 threonine (T) sites, and 13 tyrosine (Y) sites (Figure 2E), indicating that the protein may have post-translational modification and play an important role in cell signal transduction. The protein has no β-sheet structure and is mainly composed of α-helix, extended chain, and random coil. The α-helix has 467 amino acids, accounting for 47.08%. The extended chain has 153 amino acids, accounting for 15.42%; the random coil has 372 amino acids, accounting for 37.05%; and these structures are dispersed in the entire protein structure (Figure 2F), indicating that the YPR1 protein has its unique structure and function.

### 3.3. Multiple Sequence Alignment and Phylogenetic Analysis of YPR1 Gene

**Figure 3:**
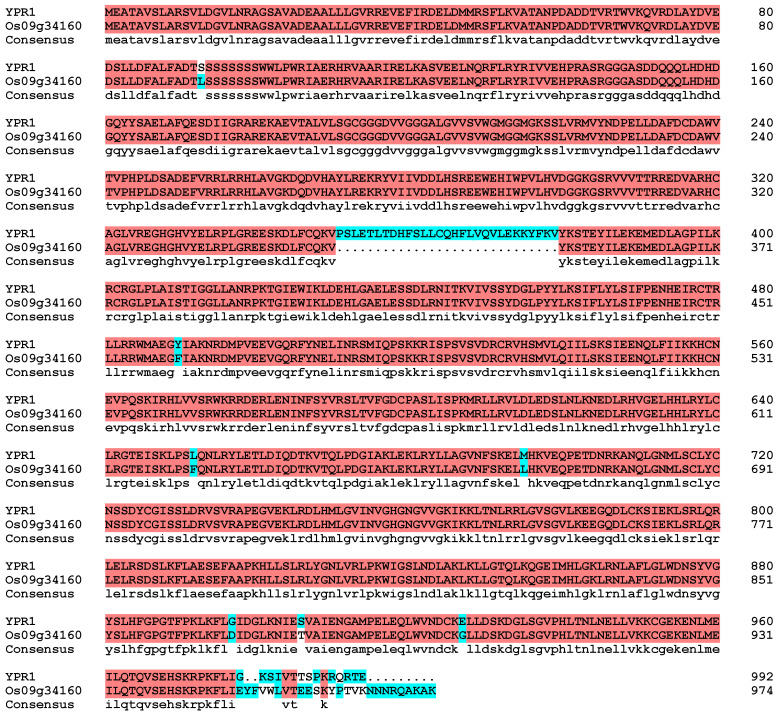
Sequence alignment analysis of YPR1 protein and Os0g34160 protein.

DNAMAN software was used for alignment analysis (Figure 3). The results showed that the amino acid sequences of YPR1 and Os09g34160 had certain differences, and the similarity between the two genes was 94.02%, indicating that *YPR1* and *Os09g34160* were not the same gene. Further, the amino acid sequence of the *YPR1* gene was input into the blastp program of the NCBI database, and it was found that the amino acid sequence of *YPR1* was not completely consistent with the amino acid sequence of any gene in NCBI database, and the similarity was only between 50.72% and 96.47%. It shows that the *YPR1* gene is a newly discovered gene and has important development and utilization value.

**Figure 4:**
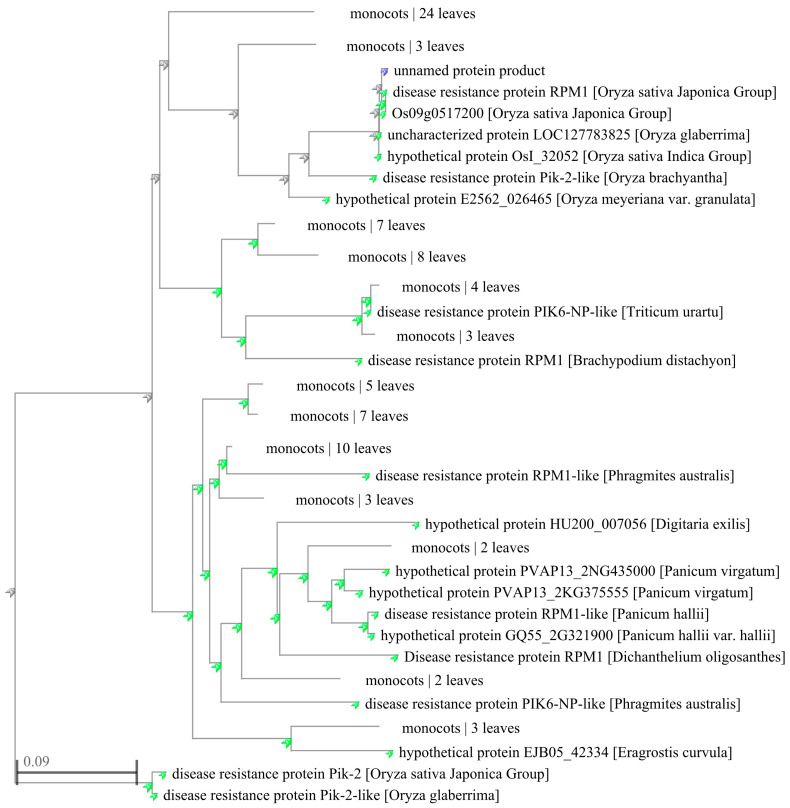
Homologous sequences alignment of YPR1 by MEGA X.

The amino acid sequences of different species with more than 50% similarity to YPR1 protein were downloaded from the NCBI database, and 100 homologous proteins were obtained, including 13 RPM1/RPM1-like proteins, 18 Pik2/Pik2-like proteins, 5 PIK6-Np/PIK6-Np-like proteins, 1 Rpp13-like protein, and proteins of unknown function. These proteins are widely distributed in rice, corn, Brachypodium, reed, wheat, sorghum, ryegrass, Miscanthus, broomcorn millet, barley, and other grasses. The phylogenetic tree (Figure 4) was constructed using the adjacency algorithm in the NCBI database. The results showed that 100 proteins could be clustered into five categories. *YPR1* was mainly clustered with RPM1 protein from japonica rice, Pik2-like protein from short anther wild rice (*Oryza brachyantha*), and eight proteins with unknown functions.

### 3.4. Analysis of Disease Resistance Function of the YPR1 Gene

#### 3.4.1. Overexpression Analysis of the YPR1 Gene

**Figure 5:**
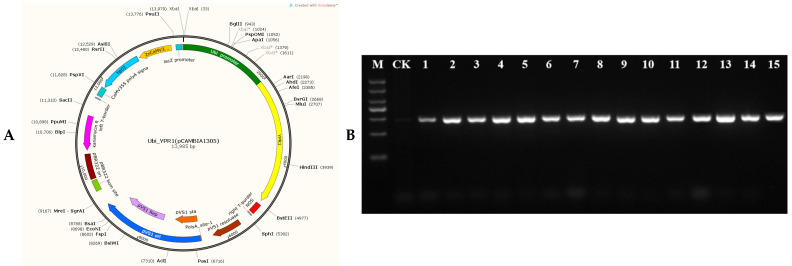

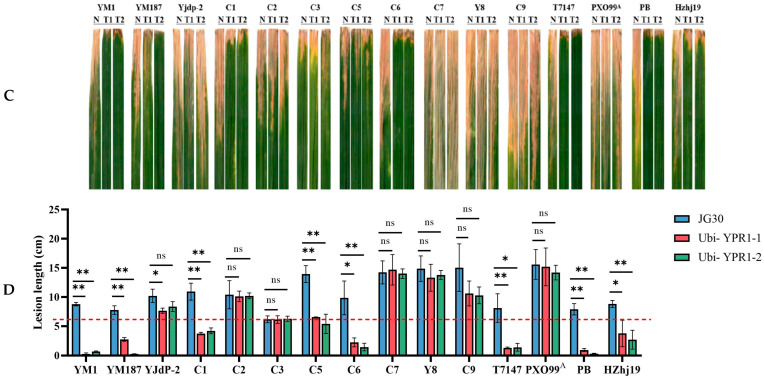
Overexpression analysis of *YPR1* gene Ubi-YPR1 overexpression vector (**A**). Transgenic positive identification of *YPR1* overexpression plants (**B**). JG30 and T1 generation of positive transgenic seedlings inoculated identification (**C**). Expression analysis of JG30 and T_1_ positive transgenic seedlings inoculated with bacteria (**D**). M: DL2000 marker, from top to bottom, are 2000 bp, 1500 bp, 1000 bp, 750 bp, 500 bp, 250 bp, and 100 bp. N is JG30 control material, T_1_ and T_2_ are overexpression materials. ^ns^ means no significant difference, * means *p* < 0.05, ** means *p* < 0.01. The red dotted line means that the anti-sense boundary is 6 cm.

In order to verify the function of the *YPR1* gene, the overexpression vector Ubi-YPR1 (Figure 5A) of YPR1 was constructed using the ubiquitin promoter, and the bacterial blight-susceptible material JG30 was transformed by the Agrobacterium-mediated method. By PCR detection of the hygromycin *Hyg* gene, 45 T_0_ generation transgenic positive seedlings were obtained (Figure 5B). The fluorescence quantitative expression analysis of the *YPR1* gene in the 45 positive transformed seedlings and the inoculation of PB strains were carried out, and 10 T_0_ generation strains with high expression and high resistance to PB were obtained. Three T_0_ generation transgenic seedlings were randomly selected, and 150 T_1_ generation seeds of each strain were selected for sowing. At the booting stage, HZhj19, PXO99 A, PB, T7147, Y8, YM1, YM187, YJdP-2, C1, C2, C3, C5, C6, C7, and C9 were inoculated with 15 strong pathogenic bacteria of bacterial blight, and each strain was inoculated with 10 transformed seedlings. At the same time, the susceptible material JG30 was used as a control, and the lesion length was investigated 15 days after inoculation. It can be seen in Figure 5C that after inoculation with Y8, YJdP-2, C2, C3, C7, C9, and PXO99A, the lesion length of susceptible material JG30 and *YPR1* overexpression seedlings was basically the same. After inoculation with 8 strains of YM1, YM187, C1, C5, C6, T7147, PB, and HZhj19, the lesion length of the susceptible material JG30 and the *YPR1* overexpression seedlings was significantly different. With 6 cm as the boundary of resistance and susceptibility, the lesion length of the susceptible material JG30 was basically above 7.75 cm (Figure 5D), while the lesion length of the *YPR1* overexpression seedlings was basically below 6.55, and even some lesions were less than 1 cm. It indicated that *YPR1* was resistant to YM1, YM187, C1, C5, C6, T7147, PB, and HZhj19 under the background of JG30, and was an important resistance gene resource.

#### 3.4.2. YPR1 Gene Editing Analysis

**Figure 6:**
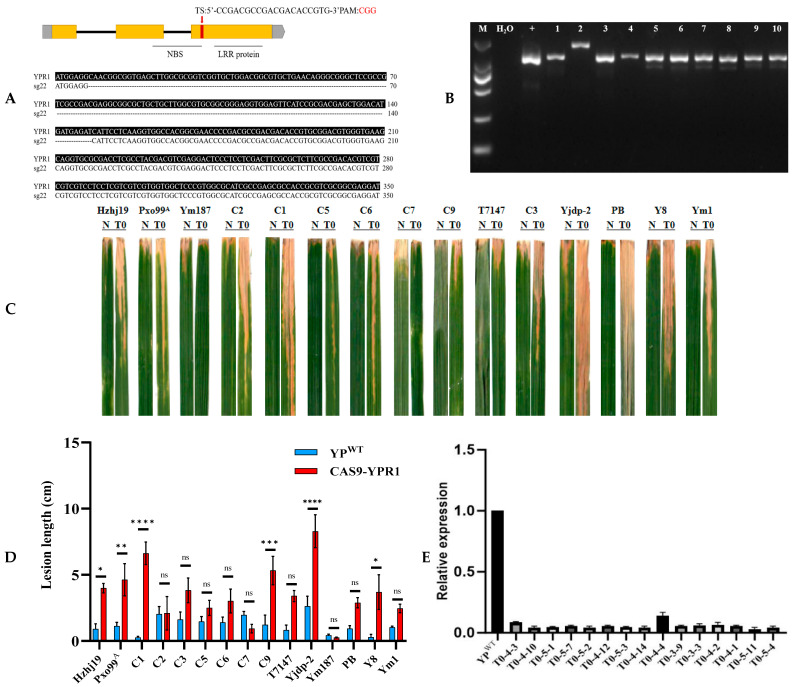
Positive knockout seedlings inoculated expression analysis Knockout target gel map (**A**). Knockout fragment sequence alignment (**B**). YP^WT^ and T0 generation positive knockout vaccine inoculation identification (**C**). YP^WT^ and T_0_ generation of positive knockout vaccine inoculation lesion length statistics (**D**). YP^WT^ and T0 generation of positive knockout vaccine inoculation expression analysis (**E**). Lesion lengths were measured 15 days post-inoculation. Data are presented as mean ± standard error (s.e.m.) from three independent biological replicates (*n* = 3), with each replicate consisting of measurements from 10 plants per strain. Statistical significance was determined by Student’s *t*-test comparing each overexpression line to the JG30 control for each strain: ** ns, not significant; *, *p* < 0.05; **, *p* < 0.01; ***, *p* < 0.001; ****, *p* < 0.0001. Gene expression was analyzed by qPCR normalized to the actin gene. Data are presented as mean ± s.e.m. from three biological replicates (*n* = 3), each with three technical replicates. Statistical significance was determined by one-way ANOVA with Tukey’s post hoc test.

The DNA sequence of the *YPR1* gene was analyzed by CRISPRP2.0 online software, and sgRNAs conforming to GN20GG or N20GG PAM sequences were screened out, and sgRNAs with target efficiency scores greater than 0.5 and high specificity were selected as candidate sequences. Finally, an sgRNA of sg22 was obtained, and sg22 was verified in vitro. Sequence alignment analysis of the target region by Sanger sequencing showed that sg22 began to have a 141 bp fragment deletion from the 8th base sequence to the 148th base sequence of *YPR1* (Figure 6A). The results showed that sg22 showed a good knockout effect. In this study, the CRISPR/Cas9 *YPR1* vector was constructed by the method established in the laboratory, and the vector was transformed into YPR1 donor parent-Yuanjiang common wild rice by the Agrobacterium-mediated method, and 15 successful knockout materials were obtained (Figure 6B). The transformed plants were detected by PCR with 35s-F/R primers, and the 15 successfully knocked out materials were positive transgenic plants.

At the booting stage of T0 generation positive gene knockout seedlings, 15 knockout materials were inoculated with 15 strong pathogenic bacteria of bacterial blight, and the disease-resistant material Yuanjiang common wild rice (YP^WT^) was used as a control. After 15 days of inoculation, the lesion length was investigated, and it was found that the knockout material showed white and yellow lesions on the leaves after inoculation, and expanded downward from the leaf tip (Figure 6C). Except for C7 and YM187, the symptoms of all knockout plants were higher than those of wild-type materials (Figure 6D). After inoculation with pathogens, the gene expression level of the knockout lines was lower than that of the wild type (Figure 6E), indicating that the function of the gene was related to the response of pathogens, and the gene knockout did successfully reduce its expression level. Through the expression level, it also shows that after the gene is successfully knocked out, the expression of its mRNA is reduced, which further verifies the effectiveness of gene knockout. The analysis of this result further showed that the *YPR1* gene played a certain role in the resistance of Yuanjiang common wild rice to bacterial blight, and had potential breeding application value.

## 4. Discussion

### 4.1. Yuanjiang Common Wild Rice Is a Valuable Resource for Exploring Excellent Genes

Yuanjiang common wild rice, as a natural gene pool, contains abundant genetic resources, which is of inestimable value for rice genetic breeding and gene function research. In recent years, with the continuous development of molecular biology and genetic technology, a series of important progress has been made in the exploration of favorable genes in Yuanjiang common wild rice. In the field of abiotic stress resistance genes, researchers have successfully excavated a number of key genes, such as the gene *qHTH5* that controls the heat tolerance of wild rice at the heading and flowering stages [27]. The study of this gene is of great significance for improving the yield and adaptability of rice in high temperature environments. In addition, the gene *SHA1* [28] of grain shattering, the gene *LABA1* [29] of long thorn awn, the gene *OsLG1* [30] of scattered panicle type, the gene *PROG1* [31] of creeping growth characteristics, the gene *hwi-1* [32] of hybrid inferior phenotype, and the gene *Xa47* [33] of bacterial blight resistance are also the research hotspots. The fine mapping and cloning of these genes provide new genetic resources for the improvement of stress resistance and agronomic traits in rice. In particular, the *PROG1* gene is closely related to the plant type of rice, and its research has attracted the attention of scientists. As an important agronomic trait affecting rice yield, panicle type is also an important goal of rice domestication and genetic improvement. Therefore, in-depth study of the scattered panicle gene *OsLG1* in Yuanjiang common wild rice will help us to analyze the molecular mechanism of rice panicle regulation and evolution, and provide a theoretical basis for cultivating new rice varieties with high yield and high quality. The *Xa47* gene is an excellent bacterial blight resistance gene discovered and studied in our laboratory in recent years. It is the same as *YPR1* gene, which is an NLR gene, and it is different from *YPR1* in disease resistance spectrum. This finding suggests that *Xa47* and *YPR1* may play a synergistic role in the resistance of Yuanjiang common wild rice to bacterial blight. However, how these two genes work together in Yuanjiang common wild rice and their specific roles in disease resistance mechanisms still need further study.

In view of the important role of genes from Yuanjiang common wild rice in agricultural production and scientific research, it is particularly urgent to explore and study the excellent genes. In summary, Yuanjiang common wild rice is a valuable resource for exploring excellent genes, and its research is of great significance for rice genetic breeding and agricultural production. Through in-depth excavation and research of the favorable genes, it will make a greater contribution to solving the problem of global food security and improving rice yield and quality.

### 4.2. YPR1 Is a Typical CC-NLR Gene

The prediction of the structure of YPR1 protein showed that the protein contained a CC domain and was a typical CC-NLR gene. The CC domain is involved in protein–protein interaction and signal transduction, indicating that the protein interacting with YPR1 is not a transcription factor but other functional proteins, and it is speculated that it may be an NLR protein. Related studies have shown that CNL-like proteins induce local cell death by directly recognizing effectors secreted by pathogens, thereby initiating an immune response. This direct recognition mode is different from the indirect recognition mode of TNL-like proteins, which makes CNL-like proteins play a unique role in plant disease resistance mechanisms, and this recognition of pathogen effectors is mainly played by the LRR region [34]. This study found that YPR1 contains 11 LRR regions. Relevant studies have shown that the polymorphism of the LRR domain determines the specificity of the NLR gene to recognize pathogen effectors. Therefore, these 11 LRR regions confer YPR1 resistance to bacterial blight. In this study, 15 strong pathogenic strains were inoculated into its overexpression lines. The gene had high resistance to 8 strains, showing its resistance to some bacterial blight strains.

### 4.3. YPR1 May Be a Multifunctional Protein

Physicochemical properties analysis showed that YPR1 protein contains inherently disordered regions and no β-sheet structure. It is speculated that this conformation can easily lead to the conversion of its secondary structure, thus affecting its biological function; that is, it may have disease resistance in some cases. In some cases, it may also have insect resistance, even drought resistance, cold resistance, and other biological functions. Related studies have shown that NLR protein mediates the recognition of specific immune response (ETI) effectors of plants to viruses, bacteria, fungi, oomycetes, parasitic plants, and herbivores, and acts as a ‘molecular switch’ to initiate ETI resistance response [35]. Like the *Sw-5b* gene, which is resistant to tomato spotted wilt virus [36]; the *Pigm* gene for rice blast resistance [37]; the *RPP1* gene of anti-ovum [38]; and the *Bph1* gene resistant to rice planthopper [39], it has been found that NLR protein not only has the function of disease resistance, but also has the function of drought resistance [40], which indicates that YPR1 protein may be a multifunctional protein. In addition, this study also found that *YPR1*, multifunctional gene *RPM1-like* [41], rice blast resistance gene *Pik2-like* [42], and proteins with unknown functions were clustered into a small class, further indicating that *YPR1* may also have rice blast resistance and other biological functions in addition to its resistance to bacterial blight.

### 4.4. YPR1 Enhances Our Understanding of NLR Diversity in Wild Rice and Its Breeding Implications

From a breeding perspective, *YPR1* presents a promising genetic resource for several reasons. Its broad-spectrum resistance against multiple prevalent Xoo strains in China makes it immediately relevant for improving elite cultivars. As a typical CNL-type gene, YPR1 is expected to function intracellularly and can be easily transferred and expressed in different genetic backgrounds. More importantly, the fact that YPR1 was successfully overexpressed in the susceptible cultivar JG30 to confer strong resistance demonstrates its robust functionality in a cultivated genetic context without being silenced or causing deleterious pleiotropic effects. This ‘plug-and-play’ capability is crucial for its application in transgenic or marker-assisted backcross breeding. Moreover, the distinct resistance spectrum of YPR1 compared to Xa47 provides a genetic basis for designing gene pyramids. Deploying these two non-allelic NLR genes together in a single cultivar could potentially delay the breakdown of resistance by making it more challenging for the pathogen to evolve virulence simultaneously against both recognition mechanisms.

### 4.5. Study Limitations and Future Perspectives

While this study provides compelling evidence for *YPR1* as a novel NLR gene conferring bacterial blight resistance, several limitations should be acknowledged. Firstly, our understanding of the molecular mechanism remains incomplete. We have not identified the specific pathogen effector protein recognized by YPR1, which is a crucial next step for elucidating the precise recognition event that triggers immunity. Furthermore, although we predicted numerous phosphorylation sites, experimental validation of their functional role in regulating *YPR1* activity is lacking.

Secondly, while CRISPR/Cas9 knockout led to increased susceptibility, confirming the gene’s necessity, we cannot fully rule out the possibility of off-target effects or the impact of the genetic transformation process on the overall phenotype. Backcrossing the mutation into a clean genetic background could further solidify the causality.

Thirdly, our resistance assessments were conducted under controlled environmental conditions. The durability and stability of YPR1-mediated resistance across diverse field environments and against evolving X*oo* populations remain to be evaluated. Finally, the potential fitness cost, if any, associated with *YPR1* overexpression in elite cultivars needs to be assessed in agronomic performance trials.

Future research will therefore focus on the following: (1) identifying the cognate effector of *YPR1* using techniques such as effectoromics; (2) validating the protein’s subcellular localization and phosphorylation status through experimental approaches; (3) pyramiding YPR1 with other broad-spectrum R genes like *Xa47* to assess the durability of resistance; and (4) evaluating the field performance of YPR1-containing lines to fully realize its translational potential.

## 5. Conclusions

In this study, we identify and functionally characterize *YPR1*, a novel CNL-type NLR gene from common wild rice that confers broad-spectrum resistance against multiple Xoo strains. Our findings underscore the immense and underutilized value of wild rice germplasm as a reservoir of unique disease resistance alleles.

Beyond its academic significance, *YPR1* holds substantial translational potential for molecular breeding programs aimed at controlling bacterial blight. The gene’s distinct resistance spectrum, which differs from that of the well-characterized *Xa47* gene from the same wild rice accession, presents a compelling opportunity for gene pyramiding. Stacking *YPR1* with *Xa47* and/or other effective R genes in elite rice cultivars is a promising strategy to create durably resistant varieties. This approach would make it significantly more challenging for the rapidly evolving *Xoo* pathogen to overcome the multi-layered defense system.

The successful overexpression of *YPR1* in the susceptible cultivar JG30 without apparent negative effects demonstrates its compatibility and robustness in a cultivated genetic background. This paves the way for its direct deployment via transgenic approaches or, once tightly linked molecular markers are developed, through marker-assisted selection in conventional breeding. In conclusion, *YPR1* is not only a valuable subject for further mechanistic study of NLR-mediated immunity but also a practical and powerful genetic tool for engineering next-generation rice varieties with sustainable and durable resistance to bacterial blight.

## Figures and Tables

**Table 1:** Detailed information of bacterial blight strains.

Strain Name	Source
C1	China standard strain No.1 race
C2	China standard strain No.2 race
C3	China standard strain No.3 race
C5	China standard strain No.5 race
C6	China standard strain No.6 race
C7	China standard strain No.7 race
C9	China standard strain No.9 race
PXO99^A^	International standard strain, race 6 of Philippine
PB	Mutant strain of PXO99^A^
T7147	International standard strain, race 2 of Japan
Y8	Strong pathogenic strains in Yunnan, China
Hzhj19	Strong pathogenic strains in Yunnan, China
YM1	Strong pathogenic strains in Yunnan, China
YM187	Strong pathogenic strains in Yunnan, China
YJdp-2	Strong pathogenic strains in Yunnan, China

**Table 2:** Primers used in this experiment.

Primer Name	Primer Sequence (5′–−3′)	Purpose
YPQC-10F	TAAGTAGCAAGCAAAACAGC	Genomic DNA amplification
YPQC-10R	AGTTATGACCCTACTGGCTC	
YP-CDS-F	ATGGAGGCAACGGCGGTGA	CDS amplification
YP-CDS-R	TTACTCTGTTCGCTGTCGTTTGGG	
Ubi-F	GGTACCCGGGGATCCTCTAGACTGCAGTGCAGCGTGACC	Vector construction (Ubi promoter)
Ubi-R	TTGCCTCCATCTGCAGAAGTAACACCAAACAACAGG	
YP-TY-F	ACTTCTGCAGATGGAGGCAACGGCGGTG	Vector construction (*YPR1* CDS)
YP-TY-R	GGGGAAATTCGAGCTGGTCACCTTACTCTGTTCGCTGTCGTTTGG	
Seq-U3	TACCACCTCGGCTATCCACA	Sequencing verification
Seq-Cas	GACAAGGGCAGGGATTTCG	
Oligo-sg22-sp1F	GGCACCGACGCCGACGACACCGTG	Oligo sequence synthesis
Oligo-sg22-sp1R	AAACCACGGTGTCGTCGGCGTCGG	
Hyg-F	ACGGTGTCGTCCATCACAGTTTGCC	Transgene detection
Hyg-R	TTCCGGAAGTGCTTGACATTGGGGA	
Yg-YPR1-F	ATGGAGGCAACGGCGGTGA	Fluorescence quantitative PCR
Yg-YPR1-R	TTACTCTGTTCGCTGTCGTTTGGG	
YP-YPR1-22-F	CAACGGCGGTGAGCTTGG	Editing site PCR detection
YP-YPR1-22-R	GTACTGCCCATCGTGGTCG	

## Data Availability

The original contributions presented in the study are included in the article, further inquiries can be directed to the corresponding author.
